# Solid-state NMR of unlabeled plant cell walls: high-resolution structural analysis without isotopic enrichment

**DOI:** 10.1186/s13068-020-01858-x

**Published:** 2021-01-07

**Authors:** Wancheng Zhao, Alex Kirui, Fabien Deligey, Frederic Mentink-Vigier, Yihua Zhou, Baocai Zhang, Tuo Wang

**Affiliations:** 1 Department of Chemistry, Louisiana State University, Baton Rouge, LA 70803 USA; 2 National High Magnetic Field Laboratory, Tallahassee, FL 32310 USA; 3 State Key Laboratory of Plant Genomics, Institute of Genetics and Developmental Biology, Chinese Academy of Sciences, Beijing, 100101 China

**Keywords:** Solid-state NMR, Dynamic nuclear polarization (DNP), Polysaccharide structure, Cellulose, Xylan, Plant cell wall, Natural isotopic abundance

## Abstract

**Background:**

Multidimensional solid-state nuclear magnetic resonance (ssNMR) spectroscopy has emerged as an indispensable technique for resolving polymer structure and intermolecular packing in primary and secondary plant cell walls. Isotope (^13^C) enrichment provides feasible sensitivity for measuring 2D/3D correlation spectra, but this time-consuming procedure and its associated expenses have restricted the application of ssNMR in lignocellulose analysis.

**Results:**

Here, we present a method that relies on the sensitivity-enhancing technique Dynamic Nuclear Polarization (DNP) to eliminate the need for ^13^C-labeling. With a 26-fold sensitivity enhancement, a series of 2D ^13^C–^13^C correlation spectra were successfully collected using the unlabeled stems of wild-type *Oryza sativa* (rice). The atomic resolution allows us to observe a large number of intramolecular cross peaks for fully revealing the polymorphic structure of cellulose and xylan. NMR relaxation and dipolar order parameters further suggest a sophisticated change of molecular motions in a *ctl1 ctl2* double mutant: both cellulose and xylan have become more dynamic on the nanosecond and microsecond timescale, but the motional amplitudes are uniformly small for both polysaccharides.

**Conclusions:**

By skipping isotopic labeling, the DNP strategy demonstrated here is universally extendable to all lignocellulose materials. This time-efficient method has landed the technical foundation for understanding polysaccharide structure and cell wall assembly in a large variety of plant tissues and species.

## Background

The past decade has witnessed the rapid advances in multidimensional solid-state NMR (ssNMR) capabilities that have enabled high-resolution characterization of intact plant cell walls. This spectroscopic method provides a wealth of atomic-level information on the conformational structure of polysaccharides, covalent linkage patterns of matrix polysaccharides, dynamical profile and water contact, as well as cellulose-matrix packing on the subnanometer scale [1]. With a rapidly expanding territory, from eudicotyledons (*Arabidopsis thaliana*) to commelinid monocotyledons (*Zea mays*, *Brachypodium distachyon*, etc.) [2–5], from primary to secondary cell walls [6–10], and from plants to algal and fungal species [11–13], ssNMR has progressively evolved into a vital tool for characterizing carbohydrate-rich biomaterials. The molecular information of cell wall architecture can serve as the structural basis for improving the current technologies of biofuel production using lignocellulosic biomass [14].

Enriching the cell walls with NMR-active isotopes (such as ^13^C and ^15^N) is a prerequisite for measuring two- and three-dimensional (2D/3D) correlation experiments, which provides the spectral resolution required for resolving numerous carbon and nitrogen sites in cell wall polymers. Two strategies can be employed: plants can be grown in the dark, using a medium-containing ^13^C-labeled glucose [2]; otherwise, ^13^CO_2_ can be continuously supplied to the plants grown in a day–night cycle [4, 15]. Depending on the developmental stage and the tissue of interest, labeling can be time-consuming and costly. In vitro replication procedures also weaken the merit of ssNMR as an analytical technique targeting natural tissues.

The recent development of Magic-Angle Spinning Dynamic Nuclear Polarization (MAS-DNP) methods has presented a unique opportunity for circumventing these drawbacks [16–19]. MAS-DNP enhances NMR sensitivity by tens to hundreds of folds, which allows us to skip isotope enrichment and use unlabeled samples to measure 2D ^13^C–^13^C/^15^ N correlation spectra for high-resolution structural characterization [20–22]. Regarding the plant biomass, three exploratory studies have been conducted to reveal the restructuring effect of ball milling on cotton cellulose [23], the alternation of xylan conformations induced by genetic mutations of rice [24], and the compositional changes of lignin in high-S and low-S poplar [25]. With the rapid development of DNP instrumentation [26–29] and radical design [30–33], this is certainly a direction of great potential but not yet explicitly explored for plant materials.

This methodology article aims at establishing a universally applicable toolbox for characterizing polymer structure and assembly in unlabeled plant biomass. This is achieved by combining a series of DNP-enabled experiments that probe the composition and conformational structure of polymers with conventional ssNMR measurements that examine the rate and amplitudes of molecular motions. Implementation of this method will expand the ssNMR capabilities and enable high-resolution investigations of unlabeled cell walls, which, at least in part, provides a replacement and upgrade to the conventional methods that rely on ^13^C-enriched materials. Most of the structural aspects previously investigated using ^13^C-enriched samples, such as the composition, conformation, packing, and motion of cell wall polymers, can be studied using unlabeled materials via a blend of MAS-ssNMR and MAS-DNP methods (Table 1). This technical advance will eliminate the threshold that has long been impeding lignocellulose characterization, which will immediately benefit the research communities of plant biology, biomaterials, and bioenergy.

**Table 1:** Capabilities of solid-state NMR and DNP in biomass research

Samples and techniques	Polymer composition	Structural polymorph	Intermolecular packing	Molecular motion	Water contact
^13^C-labeled; MAS-ssNMR	Yes	Yes	Yes	Yes	Yes
Unlabeled; MAS-ssNMR	In part	No	No	In part	In part
Unlabeled; MAS-DNP	Yes	Yes	In part	No^a^	No^a^

Technical aspects are categorized as being fully capable (Yes), partially limited by insufficient resolution or sensitivity (In part), and infeasible or unsuitable (No)

^a^MAS-DNP is conducted at cryogenic temperature (~ 100 K); therefore, it is unsuitable for investigating molecular motions. It is also better to investigate polymer–water contacts at room temperature

## Results

### Sensitivity Enhancement by MAS-DNP

**Fig. 1.**
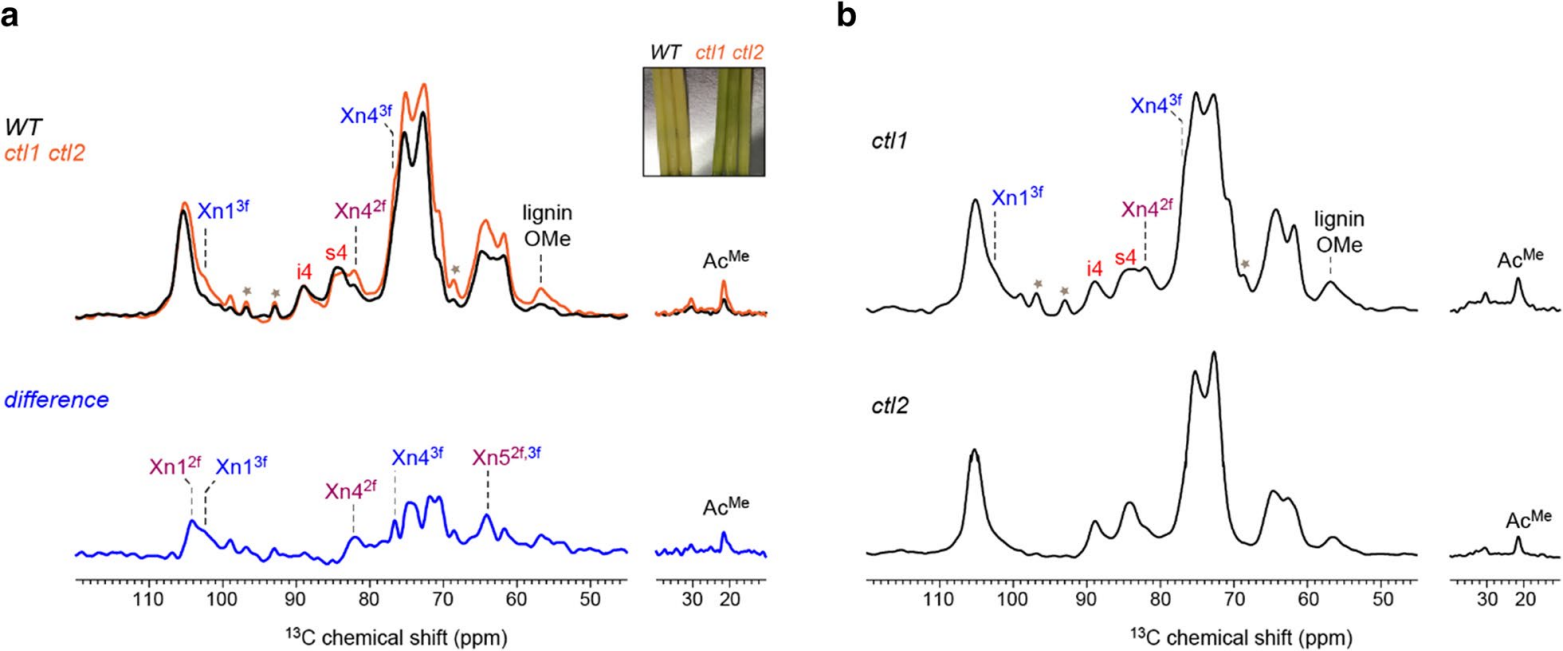
1D ^13^C spectra of unlabeled cell walls of wild-type rice and mutants at room-temperature. **a** 1D ^13^C MultiCP spectra of wild-type sample (WT; black) and *ctl1 ctl2* double mutant (orange) yielding quantitative detection of all molecules. Abbreviations of carbohydrate assignments are shown in Fig. 4, except for xylan acetyl group (Ac). The difference spectrum is obtained by subtraction of the two parent spectra after normalization by i4 peaks. The difference spectrum mainly contains xylan signals, revealing the higher hemicellulose content in *ctl1 ctl2* mutant. The insert picture on the right top side is the representative photo of the two kinds of rice stems. **b** 1D ^13^C MultiCP spectra of *ctl1* and *ctl2* single mutants. The spectral pattern of *ctl1* mutant is similar to that of the *ctl1 ctl2* double mutant while *ctl2* is closer to the WT sample

For decades, one-dimensional (1D) ^13^C spectra have been conducted using unlabeled materials to analyze the structure and composition of polysaccharides at natural isotope abundance (1.1% for ^13^C) [34–37]. Figure 1 shows the 1D quantitative spectra of four rice samples including the wild-type (WT) stems, two single mutants, which are *ctl1* (harboring an identical mutation of *brittle culm 15*) and *ctl2*, as well as a double mutant *ctl1 ctl2*. These mutations happen to rice *CHITINASE-LIKE1* (*CTL1*) and its homolog *CTL2*, which have been suggested a role in controlling cellulose biosynthesis, thereby affecting wall integrity [38–41], as *ctl1* and *ctl1 ctl2* plants exhibited obvious brittleness phenotypes. Quantitative detection of all carbons is achieved using a recently developed pulse sequence, MultiCP, which counts on ^1^H T_1_ relaxation to repeatedly restore ^1^H magnetization between the many CP blocks included in each individual scan of the experiment [42–44]. The rice stems are collected from the field, without any dissolution procedures or chemical digestions; therefore, the cell walls being analyzed are still native and intact. Various peaks of cellulose (interior chains: i; surface chains: s) and xylan (Xn) have been observed (Fig. 1a), indicating the predominance of secondary cell wall components in these mature stems.

There are ongoing debates regarding the nature of 89 and 84 ppm cellulose peaks. Recent high-resolution ssNMR studies support that these signals originate from the interior and surface glucan chains in cellulose microfibrils rather than the longitudinally distributed domains of crystalline and disordered domains [1]. The cellulose microfibrils in intact plant cell walls were found to adapt a unique structure that differs from the model Iα and Iβ allomorphs [45, 46], which were later found to only exist in highly crystalline cellulose with large crystallites, for example, the cotton balls [23]. The surface residues have extensive interactions with water molecules and matrix polymers and adapt a gauche-trans conformation, while the interior chains adapt trans-gauche conformation and exhibit substantially weaker contacts with other molecules [46–48].

Different from the WT stems, the double mutant has a higher amount of methyl ether (-OMe) groups, which is a chemical substitution frequently occurring in lignin. The *ctl1 ctl2* sample shows a higher peak at 56 ppm for methyl ether groups **(**Fig. 1a) but comparable intensities for aromatic carbons (Additional file 1: Fig. S1). This observation indicates that the *ctl1 ctl2* sample, when compared with the WT stems, has a higher fraction of the guaiacyl (G) and syringyl (S) units that contain methyl ether groups, rather than the p-hydroxyphenyl (H) unit that does not contain methyl ether group. The *ctl1 ctl2* sample has shown stronger xylan peaks, for example, the carbon 1 of threefold xylan at 102 ppm (Xn1^3f^), the carbon 4 of twofold xylan at 82 ppm (Xn4^2f^), and the methyl carbon of acetyl group at 21 ppm (Ac^Me^). Subtraction of the WT and *ctl1 ctl2* spectra has generated a difference spectrum, which contains well-resolved signals from twofold and threefold xylan, revealing a relatively higher amount of xylan (with normalization to the amount of interior cellulose) in the double mutant. The spectral patterns of the two single mutants are intriguing: the spectrum of *ctl1* resembles that of the double mutant, while *ctl2* is similar to the WT sample (Fig. 1b). Combined with the cell wall defects detected in *bc15* [41], the results have suggested a stronger role of the *ctl1* mutation in modulating cell wall composition and structure. While useful information can be obtained by following this conventional 1D analysis, only a few carbon sites can be resolved. This resolution issue can be partially alleviated by spectral subtraction, but significant improvement is still needed for characterizing these polysaccharides and cell walls.

The rice material is then impregnated in a solvent mixture of ^13^C-depleted, d_8_-glycerol/D_2_O/H_2_O, which contains 10 mM of a stable biradical AMUPol [30]. ^13^C-depletion of glycerol is a necessity for investigating unlabeled samples where both biomolecules and glycerol are at natural ^13^C abundance: it can effectively eliminate the glycerol signals that overlap with the carbohydrate signals. This protocol, when compared with a matrix-free method that was recently applied to cotton [23, 49, 50], better retains polymer hydration in cell walls. The payoff is a moderate decline in the signal-to-noise ratios, because the solvent occupies some volume in the MAS rotor, which will otherwise be filled with more biomolecules.

**Fig. 2:**
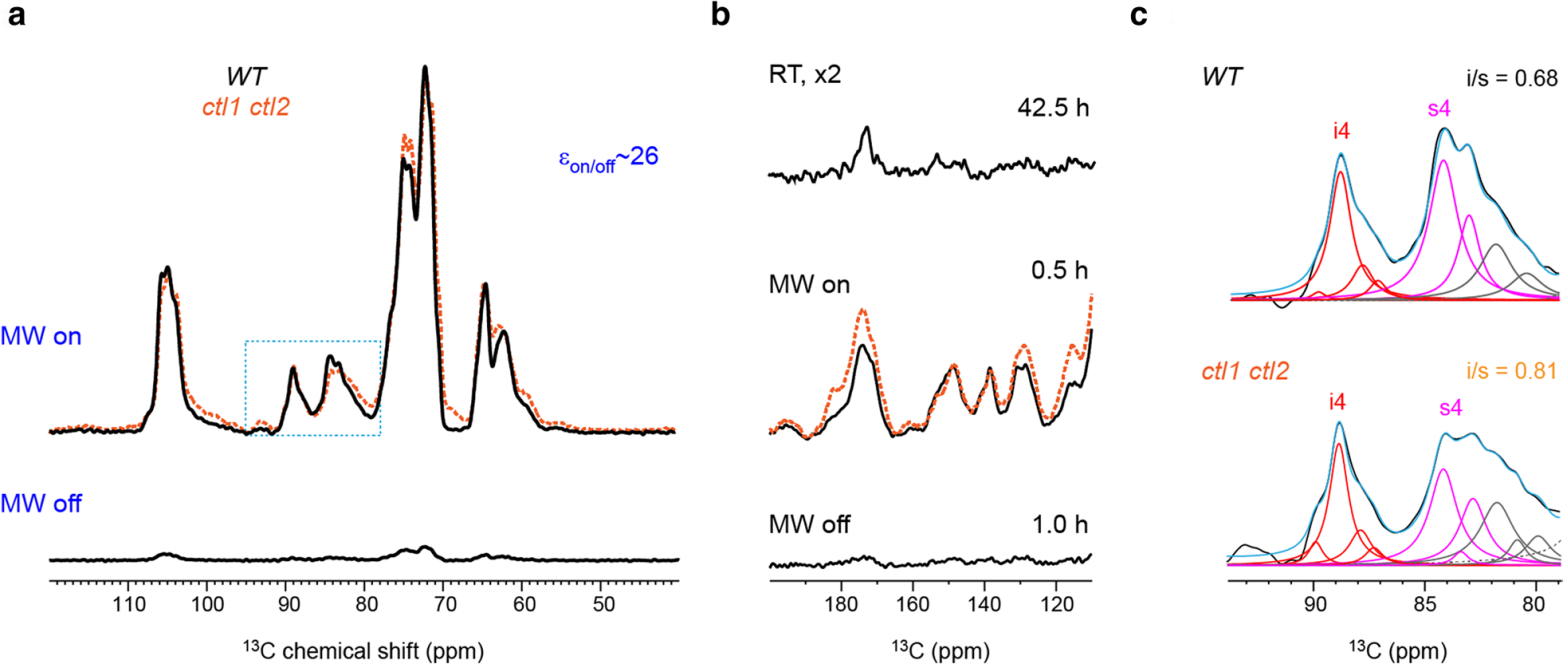
Sensitivity enhancement from DNP. **a** DNP enhances the NMR sensitivity by 26-fold on the wild-type sample. This value is obtained by quantifying the intensity ratio between the spectra collected with microwave on (MW on) and microwave off (MW off). The DNP-enhanced spectrum of *ctl1 ctl2* (orange dashline) is compared with the wild-type (WT) sample. **b** Lignin region of the room-temperature spectrum (RT; top), DNP-enhanced spectrum (MW on; middle), and non-DNP spectrum (MW off; bottom). The experimental time is labeled for each panel. DNP significantly saves experimental time and enhances NMR sensitivity. **c**, Spectral deconvolution of the interior cellulose carbon 4 (i4) and surface cellulose carbon 4 (s4) region (dashline square in panel **a**) reveals the crystallinity change in rice cellulose. The experimentally measured spectra (black) are overlaid with the simulated spectra (blue), with the deconvoluted peaks underlying the spectral envelope. The interior-to-surface (*i*/*s*) ratio of cellulose is quantified for both samples, which has shown an increase in cellulose crystallinity in the *ctl1 ctl2* mutant. The fit parameters are listed in Additional file 1: Tables S1 and S2

Under microwave irradiation, polarization of the electrons in the biradical will thereafter be transferred to ^1^H spins in unlabeled rice stem, and then to the natural-abundance ^13^C for detection. The sensitivity enhancement (*ε*_on/off_) is 26-fold for the wild-type sample and 22-fold for the *ctl1 ctl2* double mutant (Fig. 2a**,** Additional file 1: Fig. S2a), which respectively shortens the experimental time by factors 676 and 484 times. The same gain of sensitivity also occurs to lignin. We have observed strong signals of the aromatic carbons (120–160 ppm) within a short time of 0.5 h (Fig. 2b). These signals, however, are invisible in the conventional ssNMR even after 42.5 h of measurements at room temperature.

The DNP experiment preferentially detects the molecules with a highly ordered structure, for example, the cellulose microfibrils in plants (Additional file 1: Fig. S3) and the highly microcrystalline chitin in fungi [10, 12]. At the cryogenic temperature (~ 100 K) of MAS-DNP, the conformational distribution of mobile molecules, such as xylan and small molecules, will be fully trapped, resulting in broader lines and lower intensity. Recently, concerns have also been raised regarding the radical distribution in cell walls, which were supposed to induce a biased detection of molecules in spatial proximity to the radicals. This argument is invalid, because all molecules within tens of nanometer to a radical can be efficiently polarized via ^1^H–^1^H spin diffusion, which is due to the inherently long relaxation times of the ^1^Hs in cell walls [51]. This mechanism ensures a homogenous polarization of all molecules throughout the cell wall, which has been confirmed by the consistent spectral envelopes with and without microwave irradiation (Additional file 1: Fig. S4).

In addition, the crystallinity of cellulose becomes higher in the double mutant. Cellulose crystallinity is evaluated using the intensity ratio between the carbon 4 of interior glucan chains (i4; 89 ppm) and the carbon 4 of surface glucan chains (s4; 84 ppm) in cellulose. When normalized by the i4 signal, the s4 peak is lower in the double mutant than in the WT sample as consistently observed at room temperature (Fig. 1a) and under the DNP condition (Fig. 2a). Spectral deconvolution allows us to resolve the underlying resonances and quantify the interior-to-surface (i/s) ratios, which are 0.68 for WT and 0.81 for c*tl1 ctl2* (Fig. 2c**,** Additional file 1: Fig. S5**,** Additional file 1: Tables S1 and S2). The higher cellulose crystallinity in the double mutant may originate from an increased degree of cellulose bundling in the cell wall. The observation here actually counters previous X-ray analysis of *Arabidopsis*, which has shown reduced content of crystalline cellulose in a *ctl1 ctl2* mutant [40]. The discrepancy is attributed to different plant species being studied as our unpublished X-ray diffraction data on the same rice stems have observed a higher relative crystallinity index (RCI) for cellulose in the double mutant, which has confirmed the NMR observation.

**Fig. 3:**
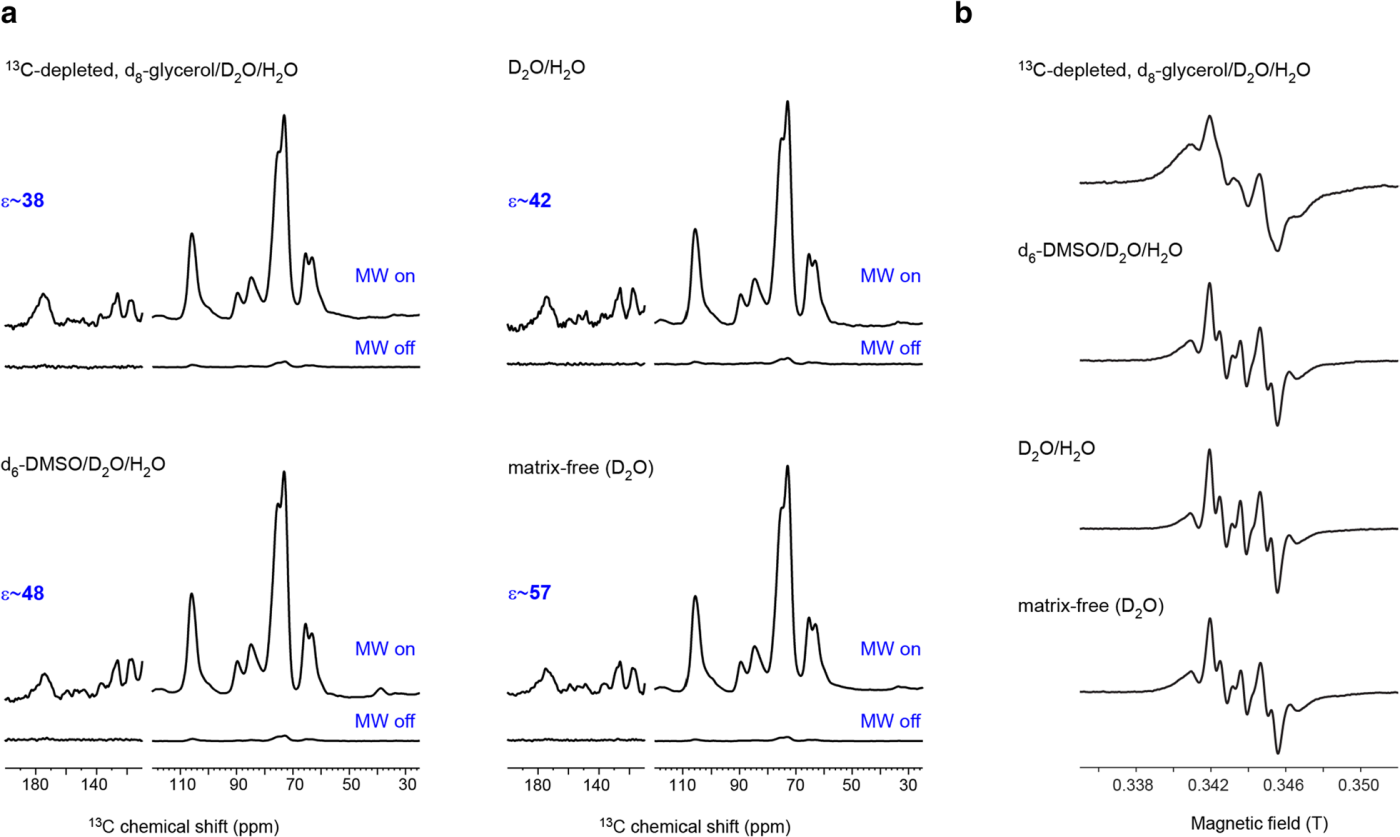
The influence of various matrices of AMUPol radical on DNP sensitivity enhancement. **a** DNP enhancement factors on the unlabeled *ctl1* sample are 38, 48, 42, and 57, respectively, when preparing the sample with ^13^C-depleted, d_8_-glycerol/D_2_O/H_2_O (60/30/10 vol%), d_6_-DMSO/D_2_O/H_2_O (80/10/10 vol%), or D_2_O/H_2_O (75/25 vol%) solvents. For the matrix-free protocol, there is no bulk solution and only a few mL of D_2_O is present in the final sample. The detailed experimental parameters are listed in Table 2. **b** Room-temperature EPR spectra of AMUPol at 9.6 GHz in the above four solvents

During the sample preparation, the radical usually is dissolved in a solvent mixture at room temperature to ensure uniform distribution. To examine how the DNP matrix affects the sensitivity enhancement, multiple DNP enhancement experiments with four major matrix protocols (glycerol/D_2_O/H_2_O, DMSO/D_2_O/H_2_O, D_2_O/H_2_O, and a matrix-free protocol) are measured. The results are shown in Fig. 3a and summarized in Table 2. The spectra of *ctl1* sample in the four solvent mixtures in Fig. 3a are highly similar, indicating that the matrix solvents have no major influence on the structure of biomolecules. Meanwhile, the matrix-free (with a limited amount of D_2_O) protocol [49, 50] gives the largest enhancement factor *ε* of 57, which means a time-saving of 3249-fold. This matrix-free protocol efficiently avoids the enhancement reduction caused by aggregation and phase separation of glass-forming solvents at 100 K.

**Table 2:** Experimental parameters of DNP enhancement

Figure number	Sample	Matrix and protocol	Rotor type	Year	Build-up time (s)	MAS (kHz)	*ε*_on/off_
Figure 2a	WT	^13^C-depleted, d_8_-glycerol/D_2_O/H_2_O (60/30/10 vol%)	ZrO_2_ thin-wall rotor	2018	2.77	10	26
Additional file 1: Fig.S2a	*ctl1 ctl2*	^13^C-depleted, d_8_-glycerol/D_2_O/H_2_O (60/30/10 vol%)			2.59	10	22
Figure 3a	*ctl1*	^13^C-depleted, d_8_-glycerol/D_2_O/H_2_O (60/30/10 vol%)	Sapphire rotor	2020	2.64	8	38
Figure 3a	*ctl1*	d_6_-DMSO/D_2_O/H_2_O (80/10/10 vol%)			1.96	8	48
Figure 3a	*ctl1*	D_2_O/H_2_O(75/25 vol%)			–	8	42
Figure 3a	*ctl1*	D_2_O (matrix-free)			1.67	8	57
Additional file 1: Fig. S2b	*ctl1 ctl2*	D_2_O (matrix-free)			1.40	10.5	44

The related figure position of each matrix protocol is given. Unidentified (–). The samples freshly measured in 2020 give high sensitivity enhancement of 38–57 fold due to the use of sapphire rotor, optimization of sample preparation, and the improvement of the DNP instruments

Besides, the second batch of samples packed in sapphire rotors and measured in 2020 have exhibited better sensitivity enhancement than the first batch of samples packed in ZrO_2_ thin-wall rotor in 2018. The reason for the improvement is multifaceted: the loss of microwave is less in the sapphire material than the ZrO_2_ material; our preparation protocol, in particular, the procedure for mixing radicals with biomaterials, has been optimized [52]; and the improved performance of the MAS-DNP instrument during the past 2 years. The continuous-wave (CW) electron paramagnetic resonance (EPR) spectra of AMUPol biradical at 9.6 GHz in the four solutions are shown in Fig. 3b. AMUPol is mostly “free” in the solution. The four spectra are similar except for the glycerol-based mixture, which is highly viscous; therefore, the tumbling rate is slow and a true liquid-state EPR spectrum cannot be observed.

### 2D ^13^C-^13^C correlation spectra of unlabeled cell walls

**Fig. 4:**
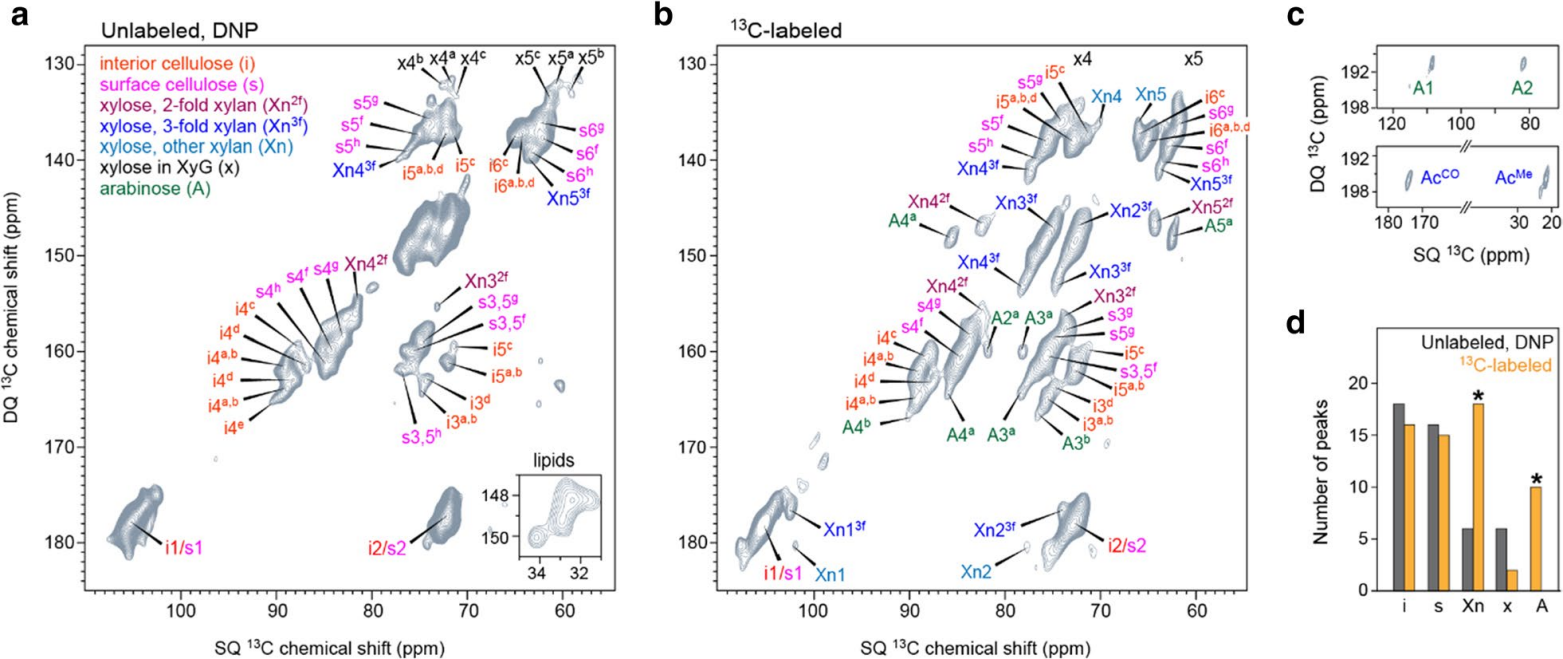
DNP enables 2D correlation spectroscopy using unlabeled and native cell walls.** a** 2D ^13^C–^13^C INADEQUATE spectrum of unlabeled WT rice collected on a 600 MHz/395 GHz DNP system. DNP effectively detects and resolves the signals from cellulose and hemicellulose at natural isotope abundance. Superscripts are used to denote the eight types of glucose units in interior and surface cellulose and the twofold and threefold conformations in xylan. Inset shows the lipid signals that are folded in the indirect dimension. **b** 2D ^13^C–^13^C INADEQUATE spectrum of ^13^C-labeled WT rice stems measured on a 400 MHz NMR spectrometer. The spectral pattern is comparable to the DNP-assisted natural-abundance spectrum in **a**. **c** Arabinose and acetyl signals are detectable using ^13^C-labeled samples, but become invisible in the DNP spectrum of unlabeled materials. **d** Number of peaks detected using labeled and unlabeled samples, which are tabulated in Additional file 1: Table S3. Asterisks indicate the components that are poorly detected using natural-abundance DNP

Benefiting from the substantial gain of NMR sensitivity, we have successfully collected a 2D ^13^C–^13^C J-INADEQUATE spectrum [53, 54] using unlabeled stems of WT rice (Fig. 4a). Because of the low abundance (1.1%) of ^13^C in nature and the even lower probability (0.01%) for observing a cross peak between two carbons, such a 2D experiment has long been deemed impossible, but can be finished now within 35 h using the rice stems.

We have observed eight types of glucose units in cellulose: types a–e for the glucan chains embedded in the core of microfibrils and types f–h for those exposed on the surface. Their ^13^C chemical shifts are consistent with those observed in *Arabidopsis*, *Zea mays,* and *Brachypodium distachyon*, revealing the polymorphic nature of cellulose structure [45]. Hemicellulose shows weak signals, for example, the carbon 4 and carbon 5 in threefold xylan (Xn4^3f^ and Xn5^3f^) as well as the carbon 3 and carbon 4 in twofold conformers (Xn3^2f^ and Xn4^2f^). In addition, the α-xylose (x) of xyloglucan also exhibits some weak signals, indicative of a small portion of primary cell walls. Besides polysaccharides, we have also observed some self-correlation cross peak of the CH_2_ acyl chains in lipid polymers. Therefore, the remarkable resolution of the DNP-enabled 2D spectrum allows us to unambiguously resolve many carbon sites in cellulose, matrix polysaccharides, and lipids, which is otherwise impossible.

The validity of natural-abundance MAS-DNP is confirmed by the consistent spectral pattern between unlabeled (Fig. 4a) and ^13^C-labeled (Fig. 4b) rice stems. In addition, a few peaks are omitted in the natural-abundance DNP spectrum. These signals are primarily from the arabinose residues of xylan sidechains, some carbon sites in twofold, threefold, and mixed forms of xylan backbones, as well as the acetyl groups (Fig. 4c). Peak counting has confirmed MAS-DNP’s preferential detection of ordered molecules: we can effectively detect all cellulose carbons and part of xylan backbones, but not for arabinose sidechains (Fig. 4d). These mobile molecules, when trapped at 100 K, bear a wide distribution of conformations, which has broadened out their signals.

**Fig. 5:**
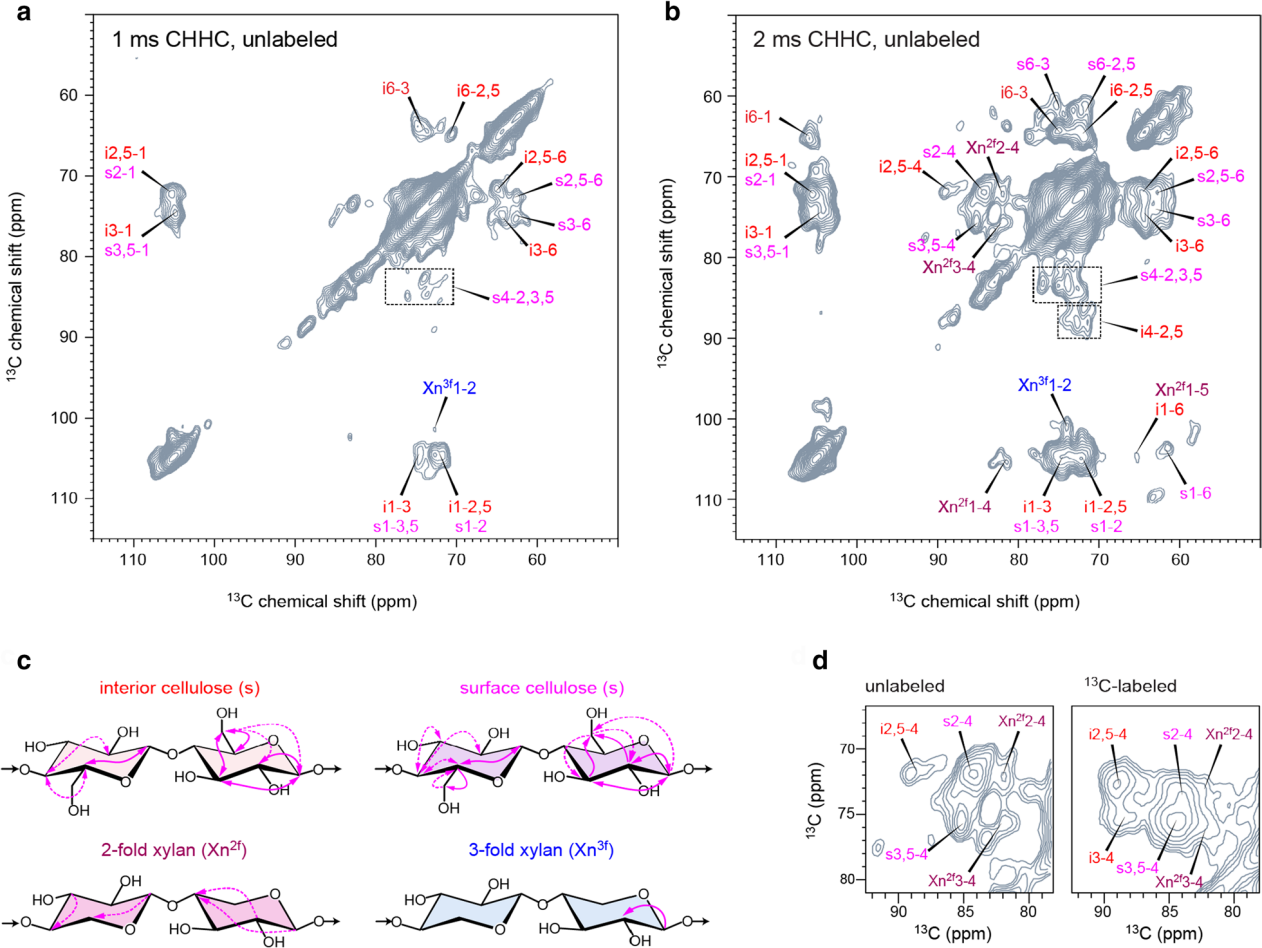
DNP-enabled 2D CHHC spectra of unlabeled rice stems. The CHHC spectra of unlabeled WT sample are measured with **a** 1 ms and **b** 2 ms ^1^H-^1^H mixing times. **c** Summary of through-space intramolecular cross peaks. Solid lines denote the cross peaks observable in both 1 and 2 ms spectra, while dash lines indicate the cross peaks that are observed only in the 2-ms spectrum. Arrows are used to indicate whether the cross peaks are bidirectional (e.g., C1-to-C2 and C2-to-C1 cross peaks) or unidirectional (e.g., only from C1 to C2). **d** Comparison of polysaccharide signals in the 2-ms CHHC spectrum of the unlabeled sample and a 5-ms PDSD spectrum of ^13^C-labeled rice

While the J-INADEQUATE experiment only probes through-bond correlations, we have also performed a CHHC experiment to detect long-range and through-space correlations (Fig. 5a, b). The CHHC sequence relies on ^1^H–^1^H spin diffusion to transfer polarization, which is followed by cross-polarization (CP) from ^1^H to ^13^C for site-specific detection [55]. The Full Width at Half Maximum (FWHM) linewidths are around 3 ppm and the representative signal-to-noise ratios are ranging from 4 to 41 (Additional file 1: Fig. S6). The off-diagonal cross peaks are mostly intramolecular correlations within cellulose, for example, the i6-3 cross peak (at 65, 75 ppm) and the s3-6 cross peak (at 75, 62 ppm) in the 1-ms CHHC spectrum. Elongating the mixing time to 2 ms allows us to extend the reach; therefore, many additional cross peaks now become observable (Fig. 5b). The new signals are primarily from cellulose, such as the i6-1 cross peak (at 65, 105 ppm) and i2,5–4 cross peak (at 72, 89 ppm). We have also observed some signature signals of xylan, for example, Xn^2f^2-4 (at 73, 82 ppm) and Xn^2f^3-4 (at 78, 82 ppm).

All the inter-carbon correlations are summarized in Fig. 5c. For cellulose, the large number of cross peaks allows us to assign the ^13^C chemical shifts of all carbons in interior and surface glucan chains. The flat-ribbon twofold xylan has shown 4 distinguishable signals, while the non-flat threefold xylan only exhibits a single cross peak from carbon 1 to carbon 2 (Xn^3f^1-2). The spectral pattern of CHHC resembles those PDSD spectra that measured using ^13^C-labeled plant biomass (Fig. 5d**,** Additional file 1: Fig. S7). Therefore, natural-abundance MAS-DNP has the full capability of investigating cellulose structure; it is also partially prepared for investigating matrix polysaccharides.

It should be noted that the CHHC experiment is specifically chosen for natural-abundance MAS-DNP. For most other 2D correlation methods, such as PDSD and DARR, the diagonal peaks will dominate the spectrum of unlabeled materials, because the probability of observing off-diagonal internuclear correlation is very low at natural ^13^C abundance. The CHHC experiment can effectively avoid this issue due to the ^13^C–^1^H–^1^H–^13^C pathway used for polarization transfer [56]. Alternatively, dipolar homonuclear recoupling schemes could also be used for characterizing unlabeled biomaterials [22, 57].

### Motional rates and amplitudes of cell wall polysaccharides

**Fig. 6:**
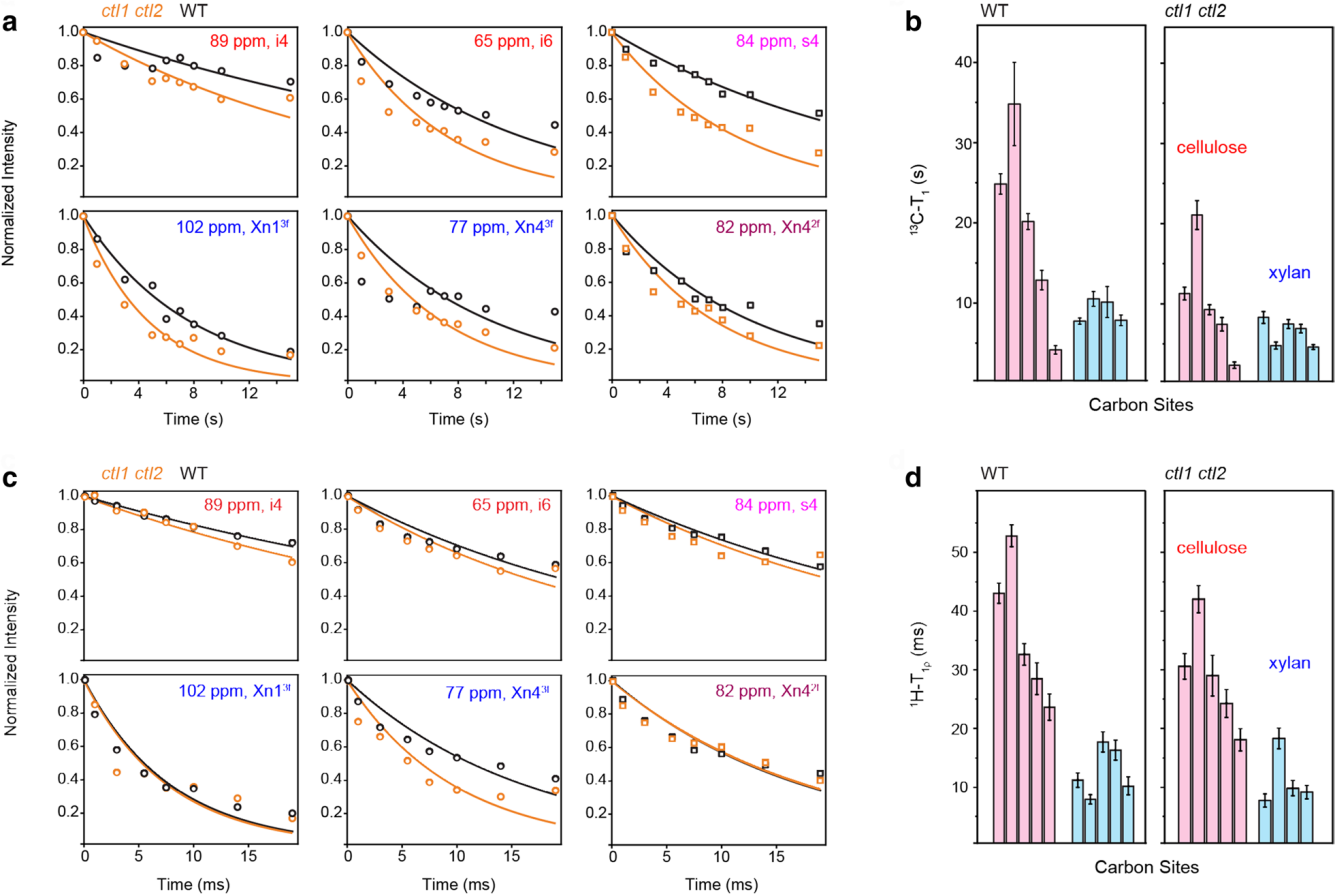
Carbohydrate dynamics probed by NMR relaxation using unlabeled rice stems at ambient temperature. **a** Representative ^13^C–T_1_ relaxation curves of cellulose (top row) and xylan (second row). The *ctl1 ctl2* double mutant has shown substantially faster relaxation than the wild-type sample, indicating more pronounced motions on the nanosecond timescale. **b** Bar diagram summarizing the ^13^C–T_1_ relaxation times of cellulose (pink) and xylan (blue). The *x*-axis represents different carbon sites, which can be found in Additional file 1: Table S4. **c** Representative ^1^H-T_1ρ_ relaxation curves. For most peaks, the double mutant shows faster ^1^H–T_1ρ_ relaxation than the wild-type sample, indicating enhanced dynamics on the microsecond timescale. All the ^1^H–T_1ρ_ relaxation time constants are summarized in **d**

To understand the effect of *ctl1 ctl2* mutation on polymer dynamics, we have measured ^13^C–T_1_ and ^1^H–T_1ρ_ relaxation times using the unlabeled stems of *WT* and *ctl1 ctl2* samples. As the most rigid component of cell walls, cellulose exhibits very slow ^13^C–T_1_ relaxation (typically on the order of 10–40 s), revealing its lack of motion on the nanosecond timescale (Fig. 6a, b, Additional file 1: Table S4 and Additional file 1: Fig. S8). In contrast, the ^13^C–T_1_ of threefold xylan, the conformer that fills the interfibrillar space, is shorter by 4–5 times: 7.7 s for carbon 1 and 10 s for carbon 4 in the WT sample. The twofold xylan, a unique form induced by its packing with cellulose surface, has shown an intermediate ^13^C–T_1_ of 10.5 s for its resolved peak of carbon 4. These ^13^C–T_1_ time constants are substantially longer than the values reported for uniformly ^13^C-labeled materials (4–6 s for cellulose and 1–2 s for matrix polysaccharides) [47]. This can be attributed to the lack of spin exchange between rigid and mobile motifs in unlabeled materials.

The ^1^H–T_1ρ_ data are highly consistent with the ^13^C–T_1_ results. Cellulose remains as the most rigid component on the slower, microsecond timescale, with a representative ^1^H–T_1ρ_ time of 20–50 ms in WT stems (Fig. 6c, d). The twofold and threefold conformers of xylan have more pronounced dynamics, showing ^1^H–T_1ρ_ values of 18 ms and 8–16 ms, respectively. Interestingly, most polysaccharides have considerably faster ^13^C–T_1_ and ^1^H–T_1ρ_ relaxations in the *ctl1 ctl2* mutant, indicative of a flawed cell wall formed by polysaccharides that are highly mobile on both nanosecond and millisecond timescales.

**Fig. 7:**
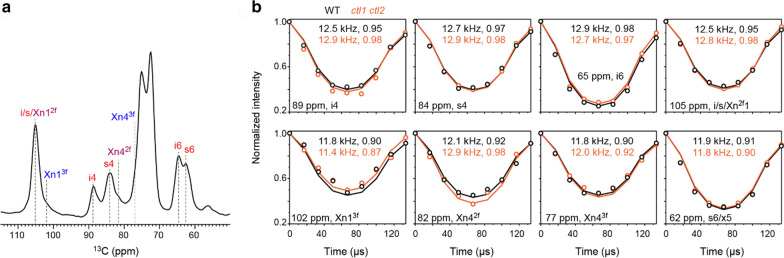
Dipolar order parameters of polysaccharides in unlabeled rice cell walls at room-temperature. **a** The first slice of the 2D DIPSHIFT experiment measured under 7.5 kHz MAS, which shows the signals of cellulose and xylan. **b** ^13^C − ^1^H dipolar evolution curves extracted from 2D DIPSHIFT experiment. The fit dipolar coupling values (in kHz) and the dipolar order parameters are labeled for each panel. Most carbon sites, except for some xylan peaks, have shown comparable order parameters in the wild-type sample and the mutant

To examine the motional amplitudes of these polysaccharides, we have conducted the ^13^C–^1^H dipolar-chemical shift (DIPSHIFT) correlation experiment at ambient temperature without DNP (Fig. 7) [58]. Polymer dynamics can only be measured using non-DNP methods (Table 1), because molecular motions will be significantly restricted at the cryogenic temperature associated with DNP experiments. The ^13^C–^1^H dipolar couplings (in kHz) are measured at representative carbon sites of cellulose and xylan, which are further converted to dipolar order parameters. A near-unity value indicates restricted motional amplitudes for the C–H bonds, while a small order parameter suggests large-amplitude motions. Strikingly, all polysaccharides are highly rigid and exhibit large order parameters above 0.90 (Fig. 7b). This is very different from the results collected using *Arabidopsis* and *Brachypodium* primary cell walls, in which the order parameters range from 0.3 to 0.6 for matrix polymers [5, 47]. Cellulose has identical order parameters in WT and *ctl1 ctl2* samples. The hemicellulose, however, has shown subtle changes: the order parameter has slightly decreased for the carbon 1 of threefold xylan (Xn1^3f^), but increased for the carbon 4 of twofold xylan (Xn4^2f^). These two peaks are the best-resolved signals of xylan, and the discrepancy observed here suggests a small extent of phase separation in the double mutant: the cellulose-packed twofold xylan becomes even more rigid in the mutant while the interfibrillar threefold conformer undergoes even larger amplitude motions. The relaxation and DIPSHIFT experiments can systematically assess the motional characteristics of polysaccharides, which complement the 2D MAS-DNP data to provide a complete view of polymer composition, structure, and dynamics in unlabeled biomass.

## Discussion

Using a common sample of agriculture lignocellulosic biomass, rice stems, we have demonstrated the feasibility of employing ssNMR and MAS-DNP methods to investigate the structure and dynamics of cell wall polysaccharides without ^13^C enrichment. The strategy and the experiments described above can be directly applied to many other biomass samples. The method is time-efficient: the total experimental time is 254 h for the wild-type stems, which includes 132 h of measurements on a 600 MHz/395 GHz DNP instrument and 122 h on a conventional 400 MHz ssNMR spectrometer. As a demonstration, this study involves the investigations of several aspects including polymer composition, structure, and dynamics. If only a single aspect is to be investigated, the experimental time will be much shorter.

Multiple factors can impact the enhancement factor and experimental time, for example, the mixing protocol that determines biradical distribution in cell wall materials, the sample pH that affects the lifetime and stability of radicals (the DNP solvent mixture by itself typically has a pH on the range of 7–8), and the choice of DNP solvent. As the beginning step, it is crucial to collect a set of 1D spectra to screen several samples prepared using different protocols and conditions, each of which only takes a few minutes to measure. By monitoring the absolute sensitivity and the DNP gain, the optimal condition will be identified for measuring 2D experiments.

At present, natural-abundance DNP still has limitations. First, it is impossible to derive a detailed pattern of intermolecular contacts using only unlabeled materials (Table 1). This is due to the demanding requirement of sensitivity for probing longer range correlations, which can even be challenging for some ^13^C-labeled samples. Progress is being made: long-range correlations, typically on the range of 3–5 Å, have been observed recently using model cyclic peptides [22]. Once successfully extended to cellular samples, these techniques will fully enable structural determination. Partial information could be obtained if the polymer contact depends on the conformational structure. For example, the flat-ribbon structure of twofold xylan is responsible for coating cellulose surface; therefore, we have tracked its amount to estimate the extent of xylan–cellulose packing in unlabeled rice stems [24]. Second, molecules with a high disorder suffer from intensity loss under MAS-DNP conditions. Fortunately, we still have distinguishable carbon sites for different xylan conformers, which can be used for structural and compositional analysis. In addition, ssNMR and DNP approaches provide atomic-level resolution on intact plant materials, but they are often limited in sensitivity and resolution for compositional analysis. Therefore, it is a promising strategy to combine ssNMR and traditional chemical assays to systematically investigate the composition of different polymers and the amount of each of their subtypes that are undertaking a variety of conformations and structures.

In this demonstrative study, we have achieved a satisfactory DNP performance using the rice stems: an enhancement factor in the range of 40- to 60-fold (Table 2) shortens the experimental time by 1600–3600 times. It should be noted that MAS-DNP is still undergoing revolution; tremendous efforts have been devoted to further improving its efficiency. Multiple biradicals have been developed recently, such as the AsymPolPOK family that shortens the DNP build-up time [33] and the Tinypol and TEMTRIPol-I radicals that have shown improved performance over AMUPol on high-field (18.8 and 21.1 T) DNP instruments [31, 59, 60]. The resolution improvement at high-field MAS-DNP (e.g., 800 MHz/527 GHz) will allow us to better analyze biomolecular structures, at least for the molecules bearing structural order [61, 62]. The rapidly evolving MAS-DNP technology has a great potential to bring biomass analysis to a new level of details.

## Conclusions

We have presented a strategy that integrates DNP-enabled natural-abundance 2D ^13^C–^13^C correlation experiments with room-temperature measurements of polymer dynamics to analyze unlabeled plant cell walls. Because this protocol no longer requires isotopic enrichment, it now becomes possible and time-efficient to screen a large collection of lignocellulose materials found in nature or engineered in vitro. Consequently, the large-scale and high-resolution biomass characterization will provide the structural foundation for improving the biotechnologies of biofuel production.

## Materials and methods

### Rice stem preparation

The mutant *ctl1* was generated by backcrossing a previously reported mutant *brittle culm 15* (A213L mutation in *OsCTL1*) into *Oryza sativa* cv. Nipponbare background [41]. The *ctl2* is an insertional mutant (NC2596 from rice *Tos17* insertion mutant database) with a *Tos17* insertion at the 1633 bp downstream of the *OsCTL2* (*LOC_Os08g41100*) coding sequence. The double-mutant *ctl1 ctl2* was created by crossing *ctl1* and *ctl2* and then screening out from the F_2_ progenies via molecular identification. All the WT and mutant rice plants (*Oryza sativa*) were grown in the experimental fields at the Institute of Genetics and Developmental Biology in Beijing (China). At least five mature stems from different plants of each genotype were harvested for the measurement.

### Dynamic nuclear polarization sample preparation

The unlabeled rice stems were sliced using a razor into pieces that are on the dimension of a few millimeters. The materials were mixed with a stock solution, which contains 10 mM AMUPol radical [30] and a solvent mixture of ^13^C-depleted, d_8_-glycerol/D_2_O/H_2_O (60/30/10 vol%). Around 50 mg of the rice material was impregnated in 100 μL of the AMUPol solution and then ground for 20–30 min, which allows the radicals to penetrate the porous cell walls. Around 33 mg of the plant material was transferred to a 3.2-mm sapphire rotor for DNP experiments. To evaluate the effect of different solvents on DNP efficiency, various matrix protocols are used, including the d_6_-DMSO/D_2_O/H_2_O (60/30/10 vol%), d_6_-DMSO/D_2_O/H_2_O (80/10/10 vol%), D_2_O/H_2_O (75/25 vol%), and a matrix-free protocol [23, 49]. A video protocol can be found in reference [52].

### Dynamic nuclear polarization experiment

All DNP experiments were conducted on a 600 MHz (14.1 T)/395 GHz MAS-DNP instrument at National High Magnetic Field Laboratory, Tallahassee, Florida [27]. The DNP spectra were collected using a 3.2-mm probe under 8 kHz MAS. All ^13^C chemical shifts were reported on the TMS scale. The microwave irradiation is around 12 W. The temperature was 104 K with microwave irradiation and decreased to 94 K when the microwave was turned off. The DNP build-up time was measured to be 2.77 s for the wild-type sample and 2.59 s for the *ctl1 ctl2* sample. Therefore, the recycle delay was typically 3.7 and 3.4 s for the wild-type and *ctl1 ctl2* samples, respectively. 1D ^13^C CP spectra were collected using a 1 ms contact time. A total number of 256 and 512 scans were collected on the wild type, and *ctl1 ctl2* samples, respectively. The total experimental time ranges from 16 to 29 min for each sample.

Two types of 2D ^13^C–^13^C correlation experiments were conducted on the rice stems: a 2D CP J-INADEQUATE experiment that probes through-bond correlations [53] and a 2D CHHC experiment that probes spatial correlations [55]. For the INADEQUATE spectra, a total number of 320 scans were recorded within 35 h for the wild-type sample. The indirect dimension includes 110 points. CHHC spectrum was much more time-consuming and was only collected on the wild-type sample. For 1 ms CHHC, a total of 320 scans were collected over 36 h, with 64 points in the indirect dimension, and for 2 ms CHHC, a total of 608 scans were collected over 60 h, with the indirect dimension varying from 50 to 60 points. The CHHC experiment relied on 3 CP blocks to transfer polarization from ^1^H to ^13^C, back to ^1^H to enable ^1^H spin diffusion, and then back to ^13^C for site-specific detection. The corresponding contact times of the three CP blocks were 1 ms, 0.5 ms, and 0.5 ms, respectively.

### Room-temperature solid-state NMR

Multiple experiments were conducted at room temperature to compare with the DNP data. Around 65 mg of unlabeled rice stems were packed into a 4-mm ZrO_2_ rotor for measurements. All non-DNP experiments were conducted on a 400 MHz (9.4 T) Bruker spectrometer using a 4-mm HCN probe. 1D ^13^C quantitative spectra were collected using the recently developed MultiCP pulse sequence at room temperature under 10 kHz [43]. This experiment allows for the quantitative detection of all ^13^C signals with enhanced sensitivity. Within each experiment, 8 CP blocks were used for WT and *ctl1 ctl2* double-mutant sample, and 9 blocks were used for *ctl1* and *ctl2* single mutant rice, with a z-filter time of 0.9 s between two CP blocks for repolarization. In total, 20 k, 23 k, 23 k, and 20 k scans were collected for the WT, *ctl1*, *ctl2*, and *ctl1 ctl2* samples, respectively. To investigate the molecular motion of polysaccharides in unlabeled rice stems, we have measured ^13^C–T_1_ and ^1^H–T_1ρ_ relaxation. ^13^C–T_1_ probes the molecular motions characteristic to the nanosecond timescale and ^1^H–T_1ρ_ probes the motion happening on the wloser, microsecond timescale. ^13^C–T_1_ was measured using a CP-based experiment [63], with a variable z-filter from 0 to 16 s. ^1^H–T_1ρ_ was measured using a spinlock field of 62.5 kHz on the ^1^H channel, with a variable spinlock time from 0 to 20 ms. The relative intensity of each data point (relative to the first data point) was plotted as a function of time and fit using a single-exponential equation. For ^13^C-T_1_ relaxation, 4 k and 3 k scans are collected for each time point of WT and *ctl1 ctl2* samples, respectively. Meanwhile, 6 k (WT) and 2 k (*ctl1 ctl2*) scans are collected for each time point of the ^1^H–T_1ρ_ relaxation experiment. In addition, we have conducted the dipolar-chemical shift (DIPSHIFT) correlation experiment under 7.5 kHz MAS [58]. Frequency-Switched Lee Goldburg (FSLG) sequence was used for ^1^H homonuclear decoupling, with a transverse field of 83 kHz and an effective field of 102 kHz. The scaling factor is 0.577 as verified using a model tripeptide MLF.

In addition, we have measured a series of 2D PDSD experiments using ^13^C-labeled rice stems to compare with the DNP-assisted 2D CHHC experiment collected on unlabeled samples. Around 85 mg ^13^C-labeled rice material was packed into a 4-mm ZrO_2_ rotor for the solid-state NMR experiment. The mixing times were chosen to be 1, 2, 3, and 5 ms for four different spectra.

### Spectral deconvolution

Spectral deconvolution of the cellulose peaks was performed using DmFit software [64]. Deconvolution was performed on the 120 to 50 ppm interval to position the baseline. For the region of interest, the C4 region, deconvolution was initiated with Lorentzian lines while keeping their number to a minimum and fixing their chemical shift according to our previous studies of cellulose conformers in plants [10, 45]. This choice was deemed satisfactory, as perfectly matching the spectrum for interior cellulose, and with a small error margin for surface cellulose. For C1, C6, and C2,3,5 regions, Gaussian lines were used to allow better fit of the bases of peaks and allow the components of C2,3,5 to have minimal overlap with the region of interest (C4). Additionally, referring to previously established chemical shifts and the data indexed in a recently developed carbohydrate ssNMR database [65] provided both good agreement of major line positions relative to the 'modulation envelope' (spectrum) and fit initiation for minor components, which amplitude is too low to be even partially resolved. Finally, it has to be noted that peak intensities are manually adjusted, as automatic computation does not usually yield satisfactory results, as algorithm tend to severely broaden every peak in C4 region to compensate baseline distortions between 90 and 95 ppm.

## Supplementary Information

**Add**i**t**i**onal f**i**le 1: Table S1.** Fit parameters of ^13^C CP spectrum of wild-type (WT) sample. **Table S2.** Fit parameters of ^13^C CP spectrum of *ctl1 ctl2* double mutant. **Table S3.** Peak numbers of INADEQUATE spectra shown in Fig. 4. **Table S4.** ^13^C-T_1_ and ^1^H-T_1ρ_ relaxation times of cellulose and xylan in WT and *ctl1 ctl2* samples. **Figure S1.** Lignin has increased methyl ether substitution in the double mutant. **Figure S2.** Additional dataset of samples prepared using different protocols. **Figure S3.** Timesaving by DNP on carbohydrate signals in unlabeled rice stems. **Figure S4.** DNP polarization is uniform across the cell wall. **Figure S5.** The experimental and simulated spectra have a good match. **Figure S6.** 1D cross sections of DNP-enabled 2D CHHC spectrum. **Figure S7.** 2D PDSD spectra of ^13^C-labeled rice stems. **Figure S8.** NMR relaxation curves of polysaccharides in unlabeled rice stems.

## Data Availability

The datasets used and/or analyzed during the current study are available from the corresponding author on reasonable request.

## Abbreviations

DNP: Dynamic nuclear polarization
ssNMR: Solid-state nuclear magnetic resonance
MAS: Magic-angle spinning
PDSD: Proton-driven spin diffusion
TMS: Trimethylsilyl
WT: Wild-type
DIPSHIFT: Dipolar-chemical shift experiment
INADEQUATE: Incredible natural-abundance double quantum transfer experiment
