# Supplementary material for: Solid-state NMR of unlabeled plant cell walls: high-resolution structural analysis without isotopic enrichment
Source: Biotechnol Biofuels. 2021 Jan 7;14:14. doi: 10.1186/s13068-020-01858-x (PMC7792314; doi:10.1186/s13068-020-01858-x)
Supplement: Supplementary file 1 — Additional file 1: Table S1. Fit parameters of 13C CP spectrum of wild-type (WT) sample. Table S2. Fit parameters of 13C CP spectrum of ctl1 ctl2 double mutant. Table S3. Peak numbers of INADEQUATE spectra shown in Fig. 4. Table S4. 13C-T1 and 1H-T1ρ relaxation times of cellulose and xylan in WT and ctl1 ctl2 samples. Figure S1. Lignin has increased methyl ether substitution in the double mutant. Figure S2. Additional dataset of samples prepared using different protocols. Figure S3. Timesaving by DNP on carbohydrate signals in unlabeled rice stems. Figure S4. DNP polarization is uniform across the cell wall. Figure S5. The experimental and simulated spectra have a good match. Figure S6. 1D cross sections of DNP-enabled 2D CHHC spectrum. Figure S7. 2D PDSD spectra of 13C-labeled rice stems. Figure S8. NMR relaxation curves of polysaccharides in unlabeled rice stems. [file 13068_2020_1858_MOESM1_ESM.pdf]

Supplementary Information

Solid-State NMR of Unlabeled Plant Cell Walls: High-Resolution Structural Analysis Without Isotopic Enrichment

Wancheng Zhao[1], Alex Kirui[1], Fabien Deligey[1], Frederic Mentink-Vigier[2], Yihua Zhou[3], Baocai Zhang[3], Tuo Wang[1*]

Correspondence: tuowang@lsu.edu

[1] Department of Chemistry, Louisiana State University, Baton Rouge, LA 70803, USA

[2] National High Magnetic Field Laboratory, Tallahassee, FL 32310, USA

[3] State Key Laboratory of Plant Genomics, Institute of Genetics and Developmental Biology, Chinese Academy of Sciences, Beijing 100101, China

Table S1. Fit parameters of ^{13}C CP spectrum of wild-type (WT) sample. The peaks are classified in three groups according to their influence on the C4 region, as by integrating, interior and surface cellulose contribution is shown to respectively be 40.5% and 59.5% of the cellulose content.

δ [ppm]	Assignment	Amplitude	Width [ppm]	Integral [%]
Cellulose C4				
90.0	i4	0.4	0.8	0.1
89.0		6.2	1.2	3.3
88.0		1.7	1.2	0.9
87.3	s4	1.0	1.0	0.4
84.3		6.8	1.6	4.7
83.1		4.1	1.2	2.2
Other peaks close to cellulose C4				
81.9		2.7	1.8	2.1
80.5		1.3	1.8	1.0
76.1		13.4	2.6	10.3
Other peaks far from cellulose C4				
105.8		13.4	1.1	6.4
104.9		11.9	1.1	5.7
103.9		10.3	1.2	5.4
74.9		20.8	1.2	7.5
74.0		17.0	1.0	4.8
72.1		40.4	2.3	27.4
71.3		3.9	0.4	0.4
64.9		11.0	1.6	5.2
64.4		7.0	0.7	1.5
63.1		6.5	1.4	2.8
61.9		9.5	1.7	4.9
59.6		3.1	2.4	3.1

Table S2. Fit parameters of ^{13}C CP spectrum of *ctl1 ctl2* double mutant. The peaks are classified in three groups according to their influence on C4 region, as by integrating, interior and surface cellulose contribution is shown to respectively be 44.6% and 55.4%of the cellulose content.

δ [ppm]	Assignment	Amplitude	Width [ppm]	Integral [%]
Cellulose C4				
90.0	i4	1.2	0.8	0.4
89.0		6.2	1.0	2.5
88.0		1.8	1.2	0.9
87.4	s4	0.9	1.0	0.3
84.3		4.9	1.5	2.9
83.5		0.7	0.8	0.2
83.0		3.4	1.5	1.9
Other peaks close to cellulose C4				
81.9		3.2	1.8	2.3
81.0		1.3	1.0	0.5
80.0		1.5	1.4	0.8
76.1		13.4	2.6	11.3
Other peaks far from cellulose C4				
105.8		9.5	0.8	3.2
104.9		13.0	1.5	7.3
103.8		10.3	1.3	5.3
100.8		1.5	4.4	2.4
74.9		23.8	1.2	7.7
74.0		19.0	1.0	4.9
72.1		39.1	2.3	23.9
71.3		7.1	0.5	1.0
70.5		6.0	0.7	1.1
64.9		13.1	1.7	5.9
64.4		3.3	1.0	0.9
63.2		8.3	1.6	3.5
61.9		6.9	1.7	3.2
59.6		4.3	3.5	5.6

Table S3. Peak numbers of INADEQUATE spectra shown in Fig. 4.

	interior cellulose (i)	surface cellulose (s)	xylose in xylan (Xn)	xylose in xyloglucan (x)	arabinose (A)
WT	18	16	6	6	0
ctl1 ctl2	16	15	18	2	10

Table S4. ^{13}C-T_1 and ^{1}H-$T_{1\rho}$ relaxation times of cellulose and xylan in WT and *ctl1 ctl2* samples. The data is fit using single exponential equation $I\ (t) = e^{-t/T}$, where T could be T_1 or $T_{1\rho}$. Error bars are standard deviations of the fitting parameters. CS: ^{13}C chemical shift. Unidentified (-).

Assignments	**Rice WT**				**Rice *ctl1 ctl2* double mutant**			
	CS (ppm)	T_1(CP) (s)	CS (ppm)	$T_{1\rho}$ (ms)	CS (ppm)	T_1(CP) (s)	CS (ppm)	$T_{1\rho}$ (ms)
i/s/Xn^{2f}1	105.6	25±1	105.5	43±2	105.5	11.3±0.8	105.3	31±2
i4	89.3	35±5	89.3	53±2	89.3	21±2	89.1	42±2
s4	84.2	20±1	84.1	33±2	84.1	9.3±0.6	84.0	29±3
i6	65	13±1	64.7	28±3	65.2	7.4±0.8	65	24±2
s6	62.9	4.2±0.5	62.8	24±2	63.1	2.3±0.4	62.8	18±2
Xn-Ac^{CO}	173.7	-	173.7	11±1	174.3	8.3±0.7	174.3	-
Xn1^{3f}	102.0	7.7±0.4	102.5	7.9±0.8	102.7	4.8±0.4	101.8	8±1
Xn4^{2f}	82.2	10.5±0.9	82.2	18±2	82.2	7.5±0.6	82.2	18±2
Xn4^{3f}	77.7	10±2	78.0	16±2	77.5	6.9±0.5	78.0	10±1
Xn-Ac^{Me}	21.6	7.8±0.7	21.7	10±2	21.7	4.6±0.3	21.7	9±1

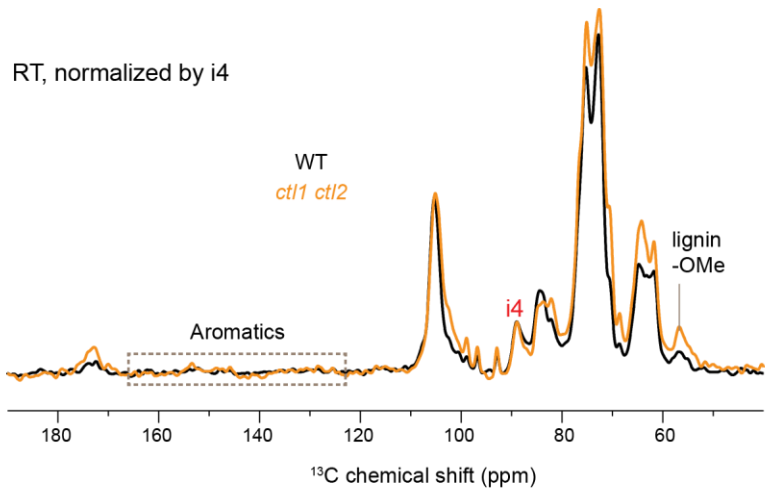

Figure S1. Lignin has increased methyl ether substitution in the double mutant. The spectra of wild-type (black) and *ctl1 ctl2* double mutant (yellow) are normalized by the interior cellulose carbon 4 (i4) peak. The lignin methyl ethers (lignin -OMe) has a doubled intensity in *ctl1 ctl2* but the lignin aromatics have a comparable intensity in both samples.

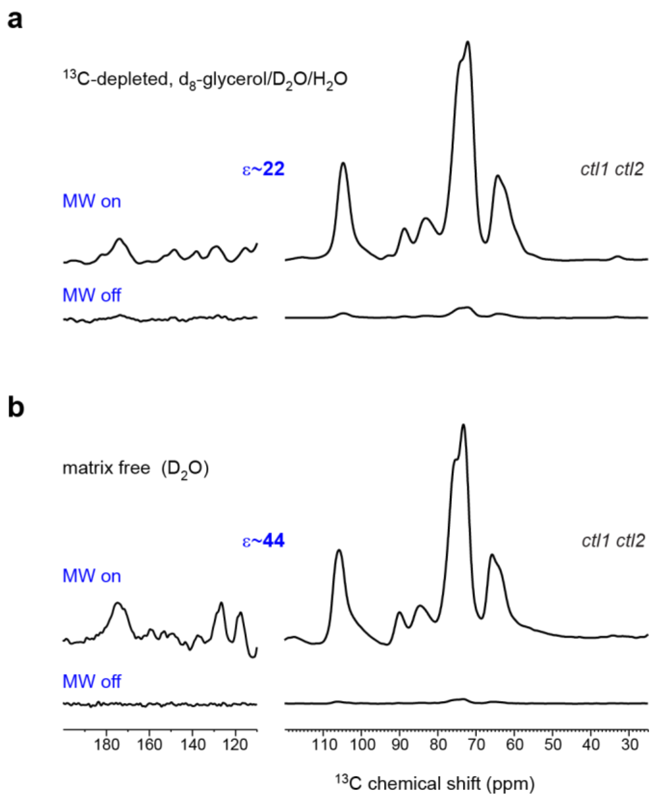

Figure S2. Additional dataset of samples prepared using different protocols. a, *ctl1 ctl2* sample in the solvent of ^{13}C-depleted, d_8-glycerol/D_2O/H_2O (60/30/10 vol%) has shown a 22-fold enhancement of sensitivity. **b**, the *ctl1 ctl2* sample prepared using the matrix-free protocol (with only a few μL of D_2O) shows an enhancement factor of 44. The detailed experimental parameters have been listed in **Table 2**.

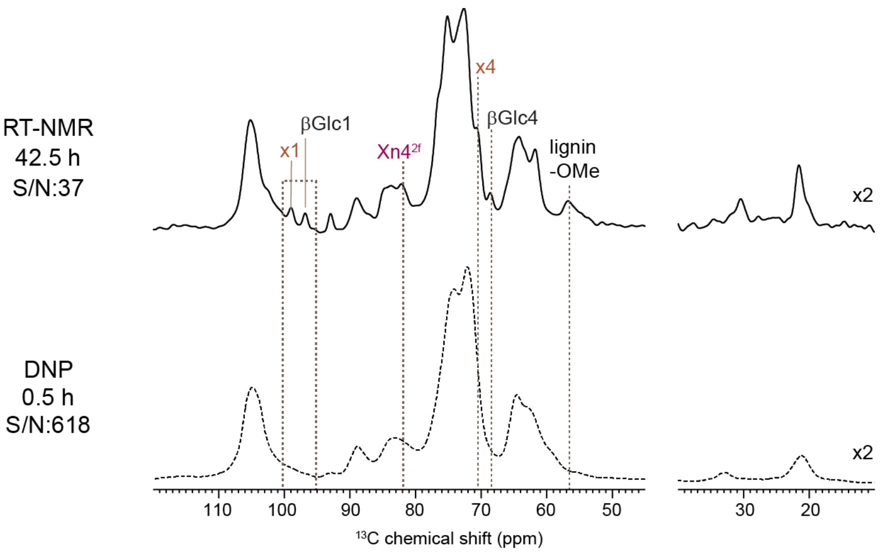

Figure S3. Timesaving by DNP on carbohydrate signals in unlabeled rice stems. Top row: The room-temperature spectrum collected on a 400 MHz NMR gives a signal-to-noise (S/N) ratio of 37 for the highest peak after 43 h of measurement. Bottom row: 600 MHz/395 GHz DNP provides a S/N ratio of 618 after only 0.5 h of measurement. Cellulose peaks are well reserved, but intensity suppression has been observed for xylan signals, lignin methyl ether (-OMe) and small molecules (Glc; glucose).

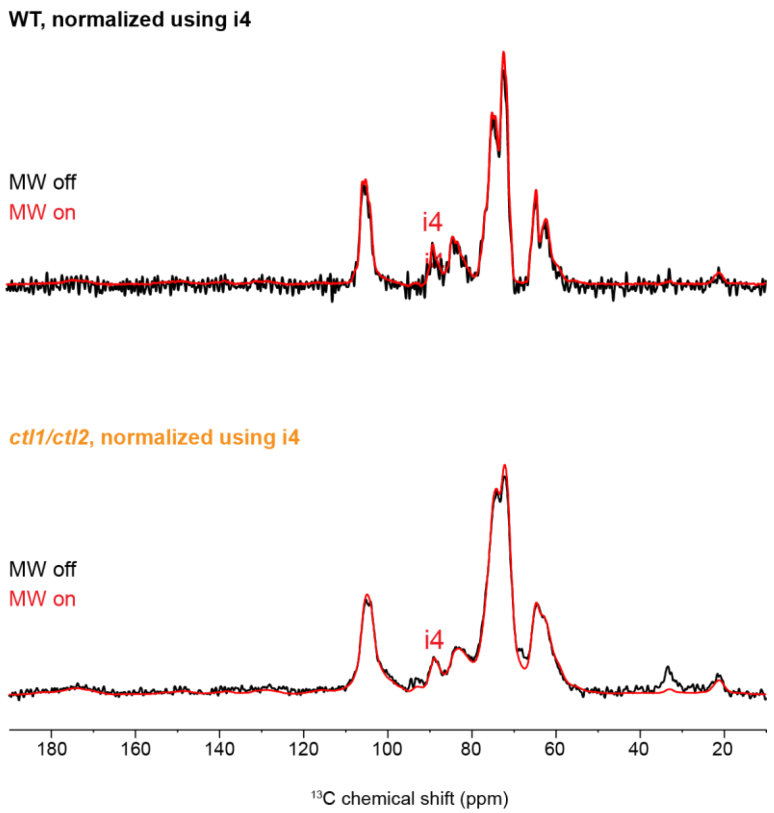

Figure S4. DNP polarization is uniform across the cell wall. The microwave-on (MW on) and microwave-off (MW off) spectra are normalized by the interior cellulose carbon 4 peaks (i4) to compare the spectral pattern. The consistent spectral envelope clearly demonstrate that the polarization is uniform across the whole cell wall.

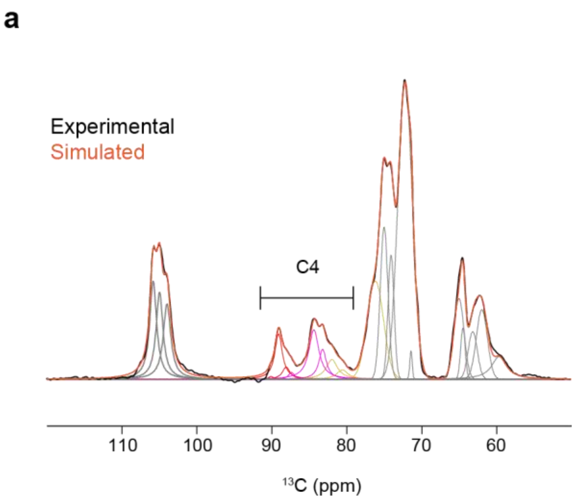

Figure S5. The experimental and simulated spectra have a good match. The 120 to 50 ppm regions of **a,** wild-type sample (left) and **b,** *ctl1 ctl2* double mutant (right) are shown. All numerical parameters used to obtain the fits are summarized in **Tables S1 and S2**. Color code follows peak classification in these tables: i4 cellulose in red, s4 in magenta, close peaks in dark yellow, and others in grey.

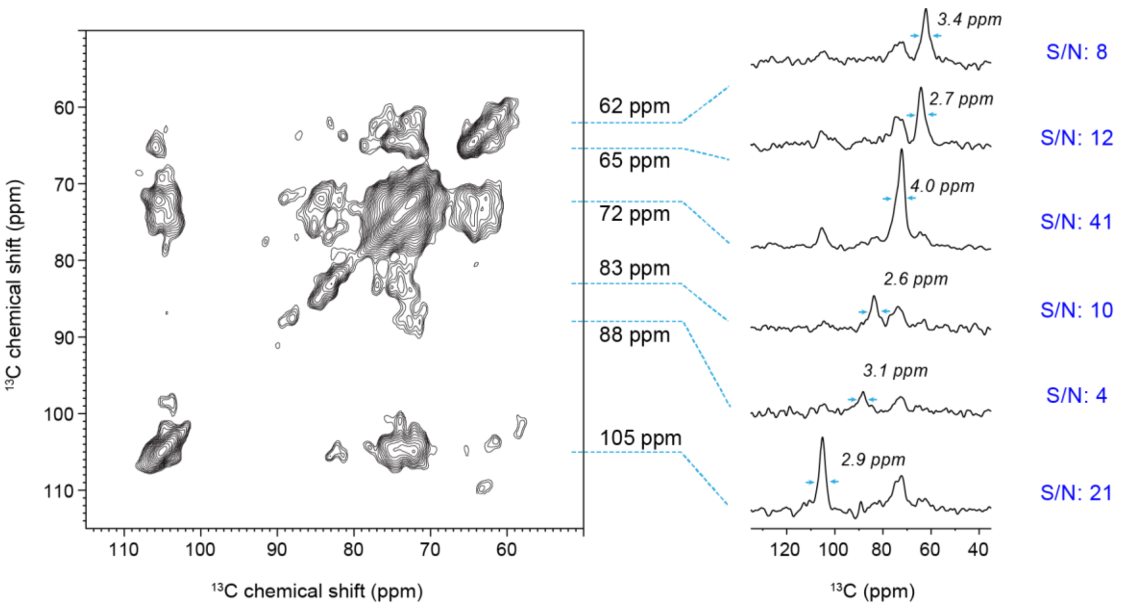

Figure S6. 1D cross sections of DNP-enabled 2D CHHC spectrum. Representative slices were extracted from the 2 ms CHHC spectrum of unlabeled wild-type rice stem. The ^{13}C FWHM linewidths and signal-to-noise (S/N) ratios are shown for the major peaks.

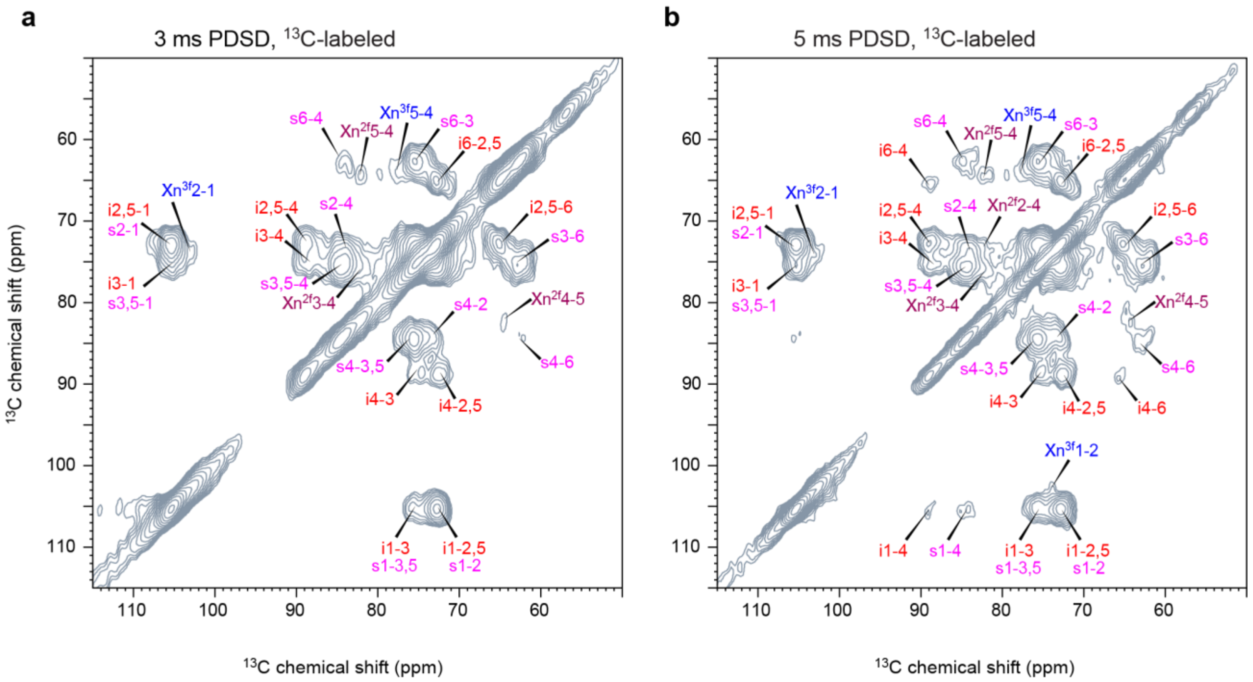

Figure S7. 2D PDSD spectra of ^{13}C-labeled rice stems. The spectra are collected using **a**, 3 ms and **b**, 5 ms mixing times. Assignments of cellulose and xylan peaks are annotated on the spectra.

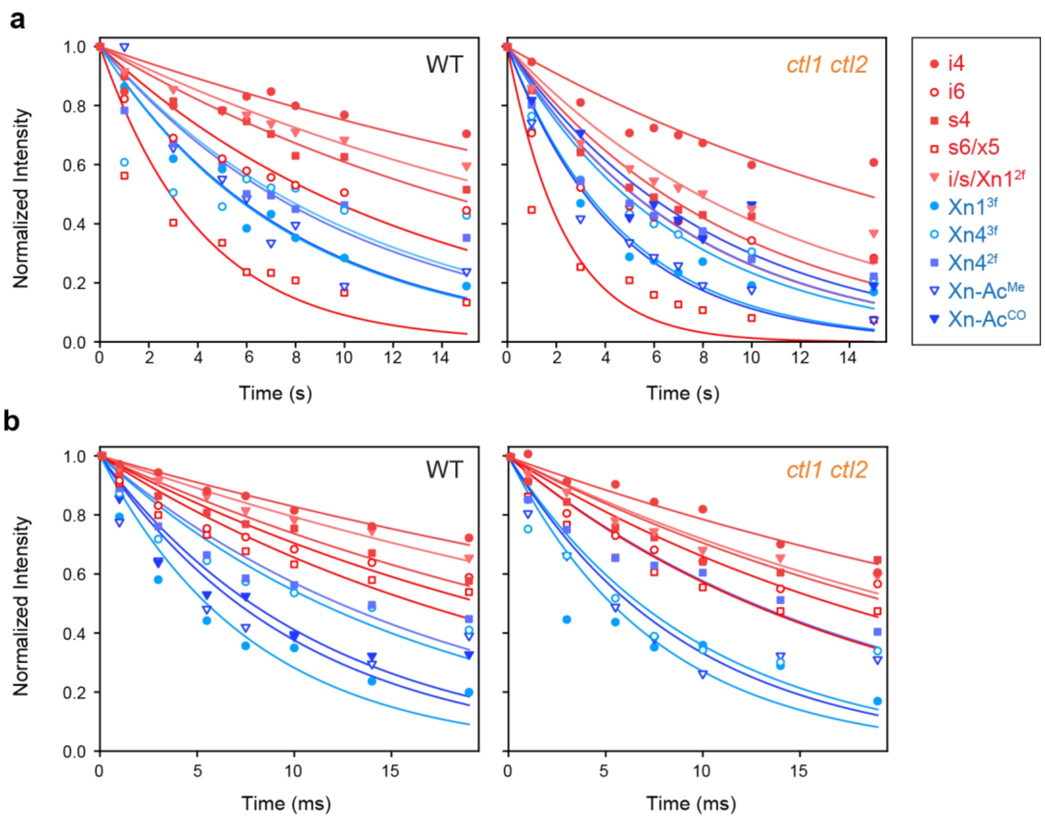

Figure S8. NMR relaxation curves of polysaccharides in unlabeled rice stems. The **a**, ^{13}C-T_1 and **b**, ^{1}H-$T_{1\rho}$ data are plotted separately for wild-type and *ctl1 ctl2* samples. Cellulose signals (red) generally exhibit faster relaxation than xylan peaks (blue). The exceptions in panel **a** only occur to the 62 ppm s6/x5 peak, which has mixed contribution from both cellulose and matrix polymers.
